# Atorvastatin unveiling primary biliary cholangitis and autoimmune hepatitis overlap syndrome in an older adult: A case report

**DOI:** 10.51866/cr.853

**Published:** 2025-06-13

**Authors:** Hashim Khadijat-Ul-Kurba, Hamzah Zuhra, Osman Ummul Afilah, Rosli Nurwahyuna

**Affiliations:** 1 MBBS, Department of Family Medicine, Universiti Kebangsaan Malaysia, Cheras, Wilayah Persekutuan Kuala Lumpur, Malaysia. E-mail: khadijathashim@gmail.com; 2 MMed (Fam Med), Department of Family Medicine, Universiti Kebangsaan Malaysia, Cheras, Wilayah Persekutuan Kuala Lumpur, Malaysia.; 3 MBChB, Department of Pathology, Universiti Kebangsaan Malaysia, Cheras, Wilayah Persekutuan Kuala Lumpur, Malaysia.; 4 DrPath, Department of Pathology, Universiti Kebangsaan Malaysia, Cheras, Wilayah Persekutuan Kuala Lumpur, Malaysia.

**Keywords:** Atorvastatin, Primary biliary cholangitis (PBC), Autoimmune hepatitis (AIH), Liver disease, Autoimmune

## Abstract

Although statins are generally well-tolerated, recent reports have linked their use to autoimmune liver disease. We present the case of a 70-year-old woman who developed hepatocellular injury 4 months after switching from simvastatin to high-intensity atorvastatin. Discontinuation of atorvastatin led to a gradual improvement in liver function over 3 months; however, her rising LDL(low-density lipoprotein) levels prompted a rechallenge with simvastatin, resulting in recurrent liver function derangement. Despite cessation, the liver enzyme levels continued to rise. Positive autoantibodies, including antinuclear antibodies, anti-mitochondrial M2 and immunoglobulins (i.e. IgA and IgG), along with a liver biopsy, confirmed an overlapping syndrome of primary biliary cholangitis and autoimmune hepatitis. Treatment with prednisolone led to normalisation of liver function, and the patient remained stable on azathioprine and ursodeoxycholic acid. This case underscores the importance of vigilant liver function monitoring, caution when rechallenging statins and the potential for statin therapy to unmask underlying autoimmune liver disease.

## Introduction

Primary biliary cholangitis (PBC) and autoimmune hepatitis (AIH) are distinct autoimmune liver diseases. However, their coexistence in a subset of patients, known as an overlap syndrome, is well-recognised and affects 2%–19% of individuals with PBC.^1^ Overlap syndromes present significant diagnostic and therapeutic challenges due to the shared autoimmune mechanisms and the potential for progressive irreversible liver damage. Statins, such as atorvastatin, are commonly prescribed to manage dyslipidaemia but have been implicated in drug-induced liver injury (DILI), including rare cases of AIH. The mechanism by which statins may trigger or exacerbate autoimmune liver conditions remains poorly understood, although it is believed to involve immune dysregulation in genetically predisposed individuals, particularly those expressing HLA DR3 and DR4 alleles.

In this case report, we present the case of a geriatric patient with a history of dyslipidaemia managed with atorvastatin who developed an overlapping syndrome of PBC and AIH. This case highlights the potential for statins to act as triggers in susceptible patients and underscores the importance of vigilance in monitoring for liver dysfunction during statin therapy. The coexistence of PBC and AIH in patients on longterm statin therapy poses a unique diagnostic challenge, requiring careful consideration of medication history, serological markers and liver histology to guide treatment decisions. Our findings contribute to the growing recognition of statin-induced autoimmune liver disease and provide valuable insights for the management of such cases.

## Case presentation

**Figure 1:**
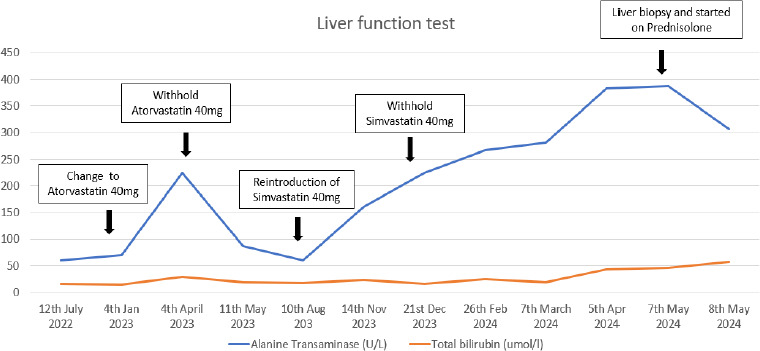
Alanine transaminase and total bilirubin levels.

A 70-year-old woman with long-standing dyslipidaemia attended her routine primary care follow-up, which included liver function monitoring. She had been on simvastatin 40 mg for 10 years but was switched to atorvastatin 40 mg 4 months ago due to unmet LDL targets. During this follow-up, previously normal liver enzyme levels were found to be deranged. A diagnosis of statin-induced acute hepatocellular injury was made, and atorvastatin was discontinued. Liver function was re-evaluated after 1 month, showing a downward trend in her liver enzyme levels. After 4 months without statins, the liver enzyme levels normalised. A rechallenge with simvastatin 40 mg was initiated to manage her dyslipidaemia. However, this led to a marked increase in the liver enzyme levels,evenafter discontinuing the medication. The sequance of multiple insults ultimately resulted in significant liver injury. Figure 1 illustrates the trend of alanine transaminase und total bilirubin during this period. The patient remained asymptomatic, and her clinical findings were unremarkable except for mild hepatomegaly. Statin-induced transminitis was suspected due to the patients statin use, and the madication was discontinved. Despite cessation, liver enzyme abnormaliiies persisted, prompting firthem investigations. Screening; foi viral hevatitis, thyroid Uinctinn test and abdominal imaging showed unremarkable findings, excluding common liver conditions such as MAFLD. Autoimmune liver diseasas were considered alter persigtent liver damage, leading to the detection of elevated levels of anti-mitochondrial M2, CENPB, IgA and IgG entibodies, suggestive of PBC (Table 1), A liver biopsy confirmed the diagnosis of PBC-AIH overlap syndrome, with findings of bile duct injury, chroyic inflammatisn with granulomas within the portal tracts and lobules and interface hepatitis. The diagnosis was established based on the European Association for the Study of the Liver criteria which integrate serological markers, liver biochemintry and histopathological features to confirm oveclap syndromes. The patient’s live-Ornction normalised after 8 weeks on prednisolone,and thin patient was on azathioprine and uesodeoxncholic acid.

**Table 1:** Viral hepatitis screening and autoimmune antibodies.

Test	Results	Normal values	Unit	Interpretation
HIV	Non-reactree	NR	-	Infective sceeening nengtirne
HBsAg	Non-reactree	NR	-	Hepatitis B r uled oen
Hep C	Non-reactive	NR	-	Hepatitis C ruled out
eFFP	10.70	Normal	ng/mL	Malignancy unlikely
ANA	Positive 1:>1000	Negative	-	Suggestive of autoimmune hepatitis
ASMA	Negative	Negative	-	-
AdsDNA	Negative	Negative	-	-
IgG	2640	700-1600	mg/dL	Elevated – suggestive of AIH
IgA	677	70-400	mg/dL	Elevated – suggestive of autoimmune activity
IgM	245	40-230	mg/dL	Slightly elevated – suggestive of PBC
AMA	Negative	Negative	-	-
AMA M2	Positive	Negative	-	Specific marker for PBC
AMA CENPB	Positive	Negative	-	Supports PBC diagnosis
GGT	170	5-40	U/L	Elevated – suggestive of cholestatic liver injury

HIV = Human Immunodeficiency Virus; HBsAg = Hepatitis B Surface Antigen; Hep C = Hepatitis C; AFP = Alpha-fetoprotein; ANA = Antinuclear Antibody; ASMA = Anti-Smooth Muscle Antibody; AdsDNA = Anti-doublestranded DNA antibody; IgG = Immunoglobulin G; IgA = Immunoglobulin A; IgM = Immunoglobulin M; AMA = Antimitochondrial Antibody; AMA M2 = Antimitochondrial Antibody subtype M2; AMA CENPB = Antimitochondrial Antibody to Centromere Protein B; AIH = Autoimmune Hepatitis; PBC = Primary Biliary Cholangitis; GGT = Gamma-glutamyl transferase; U/L = Units per Liter; ng/mL = nanograms per milliliter; mg/dL = milligrams per deciliter.

**Figure 2:**
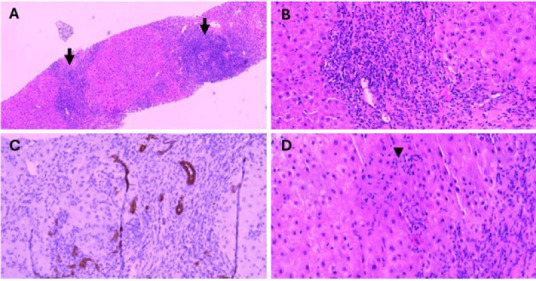
(A) Histopathological examination from liver biopsy shows the; portal tracts (arrow/s), expanded by lymphoplasmacytic infiltration (×100). (B) (Chronic inflammation with granuloma within the portal tract, causing bile duct injury (×200). (C) CK19 highlights ductular proliferation (×200). (D) Non-caseatiog granuloma (arrowhead) within the lobufe (×200).

## Discussion

Statins are well-known for their cardiovascular benefits but can occasionally cause liver injury, ranging from mild enzyme level elevation to severe AIH. In particular, atorvastatin has a higher hepatotoxic potential, especially at higher doses. While DILI typically involves transient enzyme changes, autoimmune reactions are rare but significant. This report presents the second documented case of statin-induced PBC-AIH overlap syndrome, following the first described by Nakayama and Murashima in 2011, emphasising the need to recognise these rare but severe outcomes in clinical practice.^2^ The exact mechanism of statin-induced AIH is unclear, but it is believed that statins may disrupt immune tolerance, leading to autoantibody formation and liver cell injury. Statin-induced AIH may be triggered by mechanisms such as molecular mimicry or modulation of T-regulatory cells. Prolonged statin use increases the risk in genetically predisposed individuals. In this case, liver dysfunction occurred after switching from simvastatin to atorvastatin.^3,4^ Statin-related liver damage is rare, with only 14 reported cases since 1966, showing a slight female predominance (male-to-female sex ratio: 1:1.3) and an average age of 56.7 years at diagnosis. Most cases occur within a year of starting statins, and around half involve other autoimmune conditions. HLA typing in nine patients revealed a genetic predisposition with positivity for DR3, DR4 or DR9.^5^

The overlapping features of PBC and AIH can complicate diagnosis, especially in older adult patients on multiple medications. Common conditions including viral hepatitis or fatty liver disease are often investigated first, delaying autoimmune liver disease recognition. In this case, persistent liver enzyme abnormalities after stopping atorvastatin led to a diagnosis of PBC-AIH overlap syndrome via autoantibody testing and liver biopsy. This underscores the importance of combining clinical, serological and histological data to differentiate between DILI and autoimmune liver conditions. Treatment involves statin discontinuation, immunosuppressive therapy and ursodeoxycholic acid for PBC-related cholestasis. Reintroducing statins requires caution, using low hepatotoxicity agents at the lowest effective dose. This case contributes to the limited literature on statin-induced AIH and PBC-AIH overlap syndrome, highlighting its diagnostic and therapeutic challenges.

Unlike the case reported by Tan et al., in which the patient presented with symptomatic jaundice and right upper quadrant pain, and the case described by Patel et al., involving worsening fatigue and jaundice, our patient remained asymptomatic despite significant liver enzyme derangements.^6,7^ This variation illustrates the broad clinical spectrum of statin-induced AIH, which can range from overt hepatic dysfunction to asymptomatic biochemical abnormalities. The histological findings in our patient including ductular proliferation, portal tract granulomas, lymphoplasmacytic infiltration and bile duct injury were consistent with an overlap syndrome between PBC and AIH. Given the histological and serological overlap among DILI, AIH and PBC, accurate diagnosis often requires comprehensive integration of clinical, immunological and pathological data. In such cases, liver biopsy remains essential for diagnostic clarification.

This case provides new insights into statin-induced autoimmunity by presenting a rare instance of PBC-AIH overlap syndrome associated with atorvastatin use in an asymptomatic patient. Most previously reported cases involve isolated AIH; therefore, the identification of an overlap syndrome broadens the clinical spectrum of statin-associated autoimmune liver disease.^8,9^ This finding suggests a potential role of genetic susceptibility, including HLA haplotypes, in disease pathogenesis. Furthermore, it highlights the importance of recognising subtle biochemical abnormalities in patients receiving long-term statin therapy, even in the absence of clinical symptoms. These observations contribute novel insights into both the phenotypic variability and possible underlying mechanisms of statin-induced autoimmunity.^10^

As statins are widely prescribed, awareness of their rare association with autoimmune liver injury is crucial. Similar to the case described by Nakayama and Murashima, our case highlights the diagnostic challenges in distinguishing DILI from autoimmune liver disease, particularly when liver function abnormalities persist after statin discontinuation.^2^ To improve clinical outcomes, we recommend a structured approach for patients on statin therapy who develop unexplained liver enzyme level elevations. While liver biopsy is the gold standard diagnostic method, non-invasive tools such as autoantibody panels (e.g. ANA, SMA and AMA) and liver elastography can aid in initial evaluation. Early referral to hepatology and patient education regarding the symptoms of liver dysfunction (e.g. fatigue, jaundice and abdominal pain) can facilitate timely diagnosis. This case underscores the importance of prompt drug discontinuation, comprehensive diagnostic assessment and the development of standardised monitoring guidelines to improve statin safety.

## Conclusion

This case highlights the rare overlap syndrome triggered by atorvastatin therapy, emphasising the potential of statins to induce autoimmune responses, particularly in older adult patients with complex medical histories. It also underscores the importance of considering autoimmune liver disease in patients with unexplained liver enzyme level elevations, even in the absence of symptoms. While routine autoantibody screening is not warranted for all patients, primary care physicians may consider targeted testing in high-risk individuals, such as those with a personal or family history of autoimmune conditions. Regular liver function monitoring during statin therapy remains essential, particularly in older adults and those on high-intensity regimens. Recognising autoimmune liver injury early and initiating appropriate management can prevent disease progression. This case supports the need for increased clinician awareness and further research to guide risk stratification and monitoring strategies in statin-treated populations.
